# Additive Manufacturing of Alloy 718 via Electron Beam Melting: Effect of Post-Treatment on the Microstructure and the Mechanical Properties

**DOI:** 10.3390/ma12010068

**Published:** 2018-12-25

**Authors:** Arun Ramanathan Balachandramurthi, Johan Moverare, Satyapal Mahade, Robert Pederson

**Affiliations:** 1 Department of Engineering Science, University West, SE 461 86 Trollhättan, Sweden; johan.moverare@liu.se (J.M.); satyapal.mahade@hv.se (S.M.); robert.pederson@hv.se (R.P.); 2 Department of Management and Engineering, Linköping University, SE 581 83 Linköping, Sweden

**Keywords:** Alloy 718, electron beam melting, fatigue properties, tensile properties, microstructure, texture

## Abstract

Alloy 718 finds application in gas turbine engine components, such as turbine disks, compressor blades and so forth, due to its excellent mechanical and corrosion properties at elevated temperatures. Electron beam melting (EBM) is a recent addition to the list of additive manufacturing processes and has shown the capability to produce components with unique microstructural features. In this work, Alloy 718 specimens were manufactured using the EBM process with a single batch of virgin plasma atomized powder. One set of as-built specimens was subjected to solution treatment and ageing (STA); another set of as-built specimens was subjected to hot isostatic pressing (HIP), followed by STA (and referred to as HIP+STA). Microstructural analysis of as-built specimens, STA specimens and HIP+STA specimens was carried out using optical microscopy and scanning electron microscopy. Typical columnar microstructure, which is a characteristic of the EBM manufactured alloy, was observed. Hardness evaluation of the as-built, STA and HIP+STA specimens showed that the post-treatments led to an increase in hardness in the range of ~50 HV_1._ Tensile properties of the three material conditions (as-built, STA and HIP+STA) were evaluated. Post-treatments lead to an increase in the yield strength (YS) and the ultimate tensile strength (UTS). HIP+STA led to improved elongation compared to STA due to the closure of defects but YS and UTS were comparable for the two post-treatment conditions. Fractographic analysis of the tensile tested specimens showed that the closure of shrinkage porosity and the partial healing of lack of fusion (LoF) defects were responsible for improved properties. Fatigue properties were evaluated in both STA and HIP+STA conditions. In addition, three surface conditions were also investigated, namely the ‘raw’ as-built surface, the machined surface with the contour region and the machined surface without the contour region. Machining off the contour region completely together with HIP+STA led to significant improvement in fatigue performance.

## 1. Introduction

In recent years, additive manufacturing (AM) has attracted attention from the industry and from researchers globally due to its capability to manufacture complex shaped components with relative ease compared to the conventional processing routes. Electron beam melting (EBM) is one of the powder bed fusion based technologies of AM, making use of an electron beam source to melt the metallic powder in a layer by layer fashion to produce the final component [1,2]. The process has unique capabilities for controlling the microstructural features such as texture [3] and primary dendrite arm spacing [4]. EBM offers some benefits over selective laser melting (SLM), the other powder bed fusion technology, such as the capability to control the build environment using a controlled vacuum (minimizing the risk of oxide formation during the processing) and elevated processing temperature (approximately 1000 °C for superalloys) in the build chamber which reduces the residual stresses in the built component and so forth [5]. Murr et al. compared the mechanical properties of Ti-based alloys manufactured by SLM and EBM and showed that EBM manufactured alloys exhibited superior mechanical properties (higher ductility and lower brittle phase formation) [6]. Furthermore, Antonysamy et al. reported that the fracture surface of EBM manufactured Ti-based alloys resembled the wrought alloys, which is highly desirable [7]. Therefore, EBM is a promising processing route for the manufacturing of high-performance engineering components.

Alloy 718, also referred to as Inconel-718, is a Ni–Fe based superalloy that finds application in gas turbine engine components, such as discs, blades and so forth, due to its capability to retain mechanical properties and corrosion resistance at high temperatures (650 °C). In addition, it possesses excellent formability and weldability. The phase composition of Alloy 718 predominantly consists of γ matrix along with the precipitates of γ’, γ”, δ and, in addition, some carbides (also referred to as ‘MC’s), which were also found in the EBM manufactured Alloy 718 [8,9]. These precipitates, specifically γ”, provide the desired mechanical properties to Alloy 718.

The typical microstructure of EBM manufactured Alloy 718, in the hatch region that is molten by raster scan strategy, consists of long columnar γ grains with strong <100> texture along the building direction [8,9,10,11]. Deng et al. reported that due to the spot melting strategy, the microstructure in the contour region is different from that of the hatch region [9]. The contour region has a mix of curved columnar grains, fine equiaxed grains and some large grains. In addition, it has also been demonstrated that it is possible to alter the morphology of grains, such as to vary the primary dendrite arm spacing and to attain equiaxed grain structure by carefully modifying the scanning strategy for melting [3,4,11,12,13]. The Laves phase is reported to be present in only in the top few layers; in-situ homogenization, due to the elevated powder bed temperature, leads to the dissolution of the Laves phase in the remainder of the build height [10,14]. The distribution of δ phase in an as-built part is dependent on the processing temperature, build configuration (other parts that are co-built during the manufacturing process), the cooling rate at the end of the build (from the powder bed temperature) and so forth [15,16]. Heterogeneous tensile properties along the build direction are reported to be a result of heterogeneous δ phase distribution. In addition, microstructural defects such as lack of fusion (LoF), porosity and so forth, could lead to inferior mechanical properties of Alloy 718 when manufactured with EBM [17]. The origin of defects in the EBM process has been studied in the past and was determined to be related to the process parameters [18,19].

Post-build processing of EBM manufactured Alloy 718 is a possible way to eliminate the undesirable defects (pores, LoF, cracks etc.) and phases (Laves) in the microstructure, in addition to achieving microstructural homogeneity. Most commonly employed post-processing treatments include solution treatment and aging (STA), hot isostatic pressing (HIP), machining and most often, a combination of these treatments. In case of as-cast Alloy 718, El-Bagoury et al. employed a HIP temperature in the range of 1120–1190 °C for 2 to 6 h at 200 MPa, which resulted in minimal porosity content and homogeneity of the microstructure [20]. The upper limit for the HIP temperature was chosen based on the findings of Rao et al. where exposure to temperatures exceeding 1200 °C led to grain growth [21]. Post HIP, the specimens are subjected to solutionizing treatment (typically in the temperature range of 950 °C–1050 °C and 1 to 2 h) to control δ and the Laves phase in the microstructure [22]. Schneider et al. and Chlebus et al. investigated the effect of heat treatment on the microstructure of SLM manufactured Alloy 718 and reported improvements in tensile strength and ductility compared to the as-built condition [23,24]. Similarly, Kirka et al. and Deng et al. reported improvements in the tensile properties for EBM manufactured Alloy 718 [9,25].

Research has been reported on the evaluation of the mechanical properties of EBM manufactured Alloy 718 using specimens which encompass only the hatch region and not much work has been reported the investigation of mechanical properties by retaining the contour region [9,17,25]. Antonysamy et al. [7] and Deng et al. [9] have reported the difference in grain morphology in the contour and the hatch region for Ti-64 and Alloy 718 respectively. The complete removal of the contour regions, similar to the specimens used for testing, in parts with complex geometries might not be always practical; scaling down the geometric complexity to simplify the removal of the contour region would undermine the geometric capabilities of the technology. Therefore, it is important to understand the influence of the contour region on the mechanical properties. This work aims to investigate the mechanical properties of EBM manufactured Alloy 718 specimens (by retaining the contour region) and to gain further insights into the influence of the contour region on the mechanical properties. Therefore, Alloy 718 specimens were produced by EBM and hereafter are referred to as ‘as-built’ specimens. One batch of the as-built specimens was subjected to solution treatment and ageing (STA), which will hereafter be referred to as ‘STA’ specimens. The other batch of specimens was subjected to hot isostatic pressing (HIP) followed by STA and hereafter these will be referred to as ‘HIP+STA’ specimens. The three variations, that is, as-built, STA and HIP+STA, were analysed using a light optical microscope (LOM) and scanning electron microscope (SEM) with regard to microstructure details such as defects and phases. Hardness tests were performed at different locations along the build direction (close to the build plate, at the middle of the build and near the top the build) of the specimens. The three variations were tensile tested and their fracture surfaces were analysed using SEM. Fatigue properties were evaluated only in STA and HIP+STA conditions. However, to understand the effect of the contour region, three surface conditions were investigated, namely the ‘raw’ as-built surface, the machined surface with the contour region and the machined surface without the contour region.

## 2. Materials and Methods

### 2.1. Specimen Manufacturing

**Figure 1:**
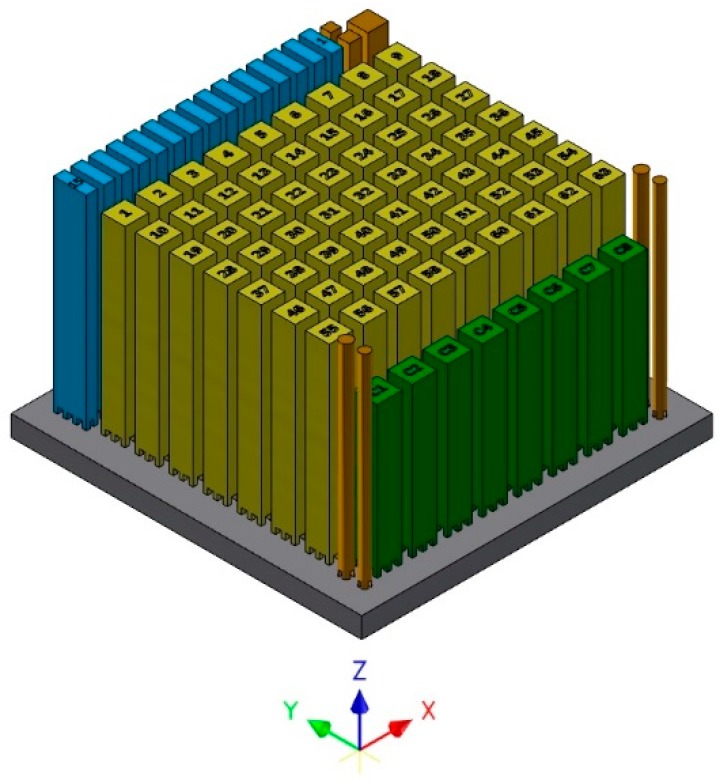
The build layout with different specimens.

An Arcam A2X machine with EBM control v4.2 was used to build 63 fatigue test specimens, 15 tensile test specimens, eight Charpy impact test specimens and seven other specimens. The fatigue test specimens built in this work had dimensions of 11.5 mm × 11.5 mm × 90 mm, whereas the tensile test specimens had dimensions of 15 mm × 5.5 mm × 95 mm. Out of the 63 specimens, 48 were used for four-point bending fatigue tests and 11 out of 15 specimens were used for tensile testing. The remaining specimens from the build were utilized for microstructural analysis or other additional tests. The build layout with all the specimens is shown in Figure 1.

**Figure 2:**
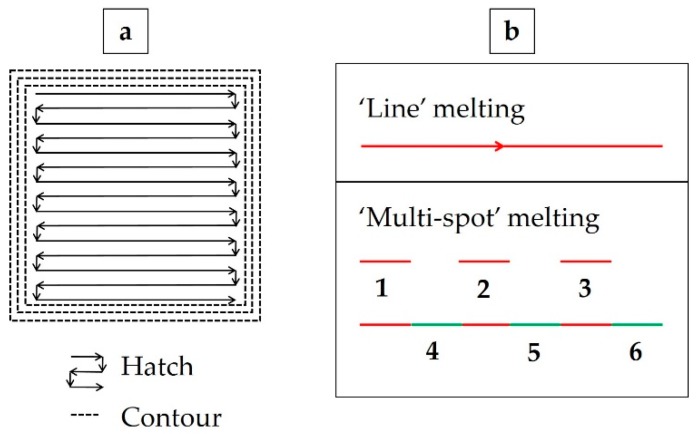
(**a**) Schematic image representing the difference in melting strategy between contour and hatch (as seen perpendicular to the layers). Note: There is overlap between the individual points and the lines as well as between the contour and the hatch in the melting strategy. (**b**) Schematic image representing ‘line’ melting and ‘multi-spot’ melting strategies.

Commercially available plasma atomized powder provided by Arcam with a chemical composition as listed in Table 1 and size range of 40–105 µm was used in this work. One single batch of virgin powder was used to build the specimens. The standard build theme, Inco 4.2.76, provided by Arcam, was utilized for printing the Alloy 718 specimens. In this theme, there are three ‘contours’ that are melted using the ‘multi-spot’ strategy. The ‘hatch’ is melted using ‘snaking’ strategy, which features one continuous line from the starting point until the end point. A schematic sketch of the EBM manufacturing strategy is shown in Figure 2a. The contour region extends up to ~1.5 mm from the periphery. The hatch region starts from ~2 mm from the periphery; between the contour and the hatch is the contour-hatch interface region. Figure 2b is a schematic to explain the ‘line’ melting and ‘multi-spot’ melting strategies for the contour. In the ‘line’ melting strategy, the electron beam is moved from one end to the other in one pass, whereas in the ‘multi-spot’ melting strategy, the melting is carried out as a sequence of line segments, from one end to the other, until the entire length is molten. The exact sequence and the length of the segments are dependent on the total length of the line to be melted (dependent on part geometry), the number of spots, speed function or beam velocity and spot time and so forth. Some of the key processing parameters utilized are listed in Table 2. All the compensation functions in the theme were used and were not altered.

After the build completion, the ‘sinter-cake’ was removed from the powder bed to recover the sintered powder using an Arcam powder recovery system. The specimens were cut off from the build plate using a band saw.

### 2.2. Metallography

Metallographic analysis of the as-built, the STA and the HIP+STA specimens was performed at three locations, including the midsection, the top and the bottom end of the fatigue bars. For the specimens in STA and HIP+STA conditions, the location identifiers were lost during machining of the specimens; hence, the top and the bottom could not be identified. The ends are denoted as ‘end 1′ and ‘end 2′ henceforth. Samples were cut, mounted, ground and polished using standard metallographic procedures. Electrolytic etching was done with oxalic acid and 3V for ~5 s to reveal the microstructure. Microstructural investigations were performed with a Zeiss AX10 light optical microscope at suitable magnifications. In addition, detailed analyses at higher magnifications were performed using Zeiss EVO 50 SEM fitted with Oxford Xmax^N^ energy dispersive X-ray spectroscopy (EDS) or a Hitachi SU70 FEG SEM, equipped with EDS and electron backscatter diffraction (EBSD) systems from Oxford Instruments. Texture analysis was performed with the scanning step size of 1–2 µm and analysed using the HKL Channel 5 software.

### 2.3. Thermal Post-Treatment

HIP was performed in a Quintus QIH21 HIP furnace at 1200 °C and 120 MPa for 4 h followed by uniform rapid quenching (URQ). STA was performed in a vacuum furnace. The solution treatment was performed at 1066 °C for 1 h and argon quenched to 65 °C. This was followed by a two-step ageing treatment at 760 °C for 10 h at 649 °C for 8 h, with furnace cooling at 55 °C/h between the two steps and argon cooled to room temperature at the end. One batch containing 24 fatigue specimens (out of 48) and four tensile specimens (out of 11) was subjected to HIP followed by STA, which are referred to as the ‘HIP+STA’ specimens. Another batch containing the remaining 24 fatigue specimens and four tensile specimens was STA treated, which are referred to as the ‘STA’ specimens. The remaining specimens were retained in the as-built condition.

### 2.4. Hardness Testing

An HMV Microhardness Shimadzu machine was used to measure Vickers hardness (HV_1_). A load of 1 kg was applied for 15 s with a suitable distance between the individual indents and from the edge of the samples. The tests were performed on cross-sections perpendicular to the build direction in as-polished samples. For each specimen, at least 15 indents were made to obtain reasonable statistics.

### 2.5. Tensile Testing

Tensile tests were conducted along the build direction on specimens in as-built condition as well as in both post-treated conditions. The specimens were machined to the conventional flat dog bone type geometry with a parallel section length of 26.8 mm and a rectangular cross section of 5.25 mm by 3 mm. The radius between the narrow section and the grip section was 10 mm. The tests were conducted in ambient conditions at a strain rate of 10^−3^/s using Instron 5582 and Instron 8802 machines; post-treated specimens were tested in Instron 5582, while the as-built specimens were tested in Instron 8802. A minimum of three specimens were tested for each test condition.

### 2.6. Fatigue Testing

**Figure 3:**
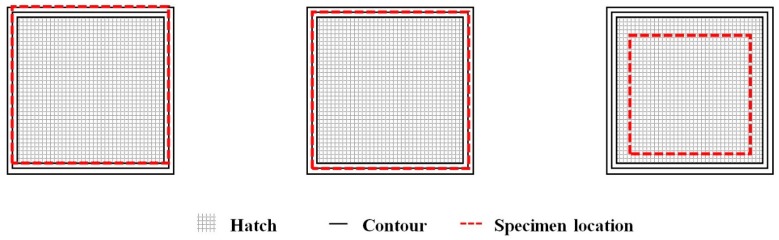
A schematic representation indicating the location of the extracted fatigue specimens from the built specimens.

Specimens for fatigue testing were prepared by machining and low stress grinding after the thermal post-treatments. Out of all the specimens used for fatigue testing, 30 were machined on all four sides, whereas 18 other specimens were machined such that three sides were completely machined and one of the sides was machined partially to retain the “raw” as-built surface. The “raw” as-built surface was retained for 40 mm in the middle of the partially machined side. This was performed to facilitate proper contact between the rollers in the test fixture and the specimens (at the machined areas). Out of the 30 completely machined specimens, 18 were machined to have 10 mm × 10 mm sides, while 12 others were machined to a smaller 6 mm × 6 mm sides. The smaller specimens were machined such that only the hatch region was present and the entire contour region was removed (see the schematic representation in Figure 3). All specimens were 80 mm long and the edges were rounded off suitably to avoid edge effects. The machined surfaces were finished to a R_a_ value of 0.2 µm. A summary of the specimen conditions is presented in Table 3.

Load controlled four-point bending fatigue tests were performed in constant amplitude mode on a servo-hydraulic test machine. The testing machine was fitted with a self-aligning four-point bending fixture, an Instron 8800 controller and a ±30 kN load cell. The tests were performed at room temperature, stress ratio R = 0.1 and 20 Hz frequency. The specimens in different thermal and surface post-treated conditions were tested with at least three different stress ranges. The as-built specimens were mounted on the four-point bending fatigue fixture such that the “raw” as-built surface was under tensile stress due to bending. Some of the results from these tests have already been published elsewhere [19].

### 2.7. Fractography

Fracture surfaces of both tensile and fatigue tested specimens were first analysed using an Olympus SZX9 stereomicroscope. Further analysis at high magnification was performed using a Zeiss EVO 50 SEM.

## 3. Results

### 3.1. Powder Characterization

**Figure 4:**
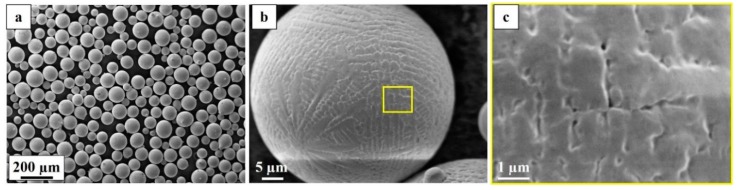
Secondary electron (SE) image of powder particles. (**a**) Low magnification image of powder particles. (**b**) High magnification image of one powder particle. (**c**) High magnification image of the area indicated in (b).

The feedstock used in this study was pre-alloyed virgin powder of Alloy 718, manufactured by plasma atomization. The powder particles were spherical with a minimal amount of satellites, as shown in the low magnification secondary electron (SE) image in Figure 4a. The high magnification SE images, Figure 4b,c, show the surface morphology of the powder that had a dendritic structure. Furthermore, the surface morphology of the powder is typical of the virgin powder without the presence of any particulate oxides.

### 3.2. Microstructure

The microstructural investigation in the present study includes characterization of the different phases and their distribution, the different defects and their distribution and finally, texture analysis. The top few layers, which have been reported to retain the solidification structure and Laves phase, are not characterized in this study, as this region does not contribute to the bulk properties of tall specimens/parts [10,14]. The results pertaining to the phases and defects for the three material conditions are presented first, followed by the results of the texture analysis.

#### 3.2.1. As-built Condition

**Figure 5:**
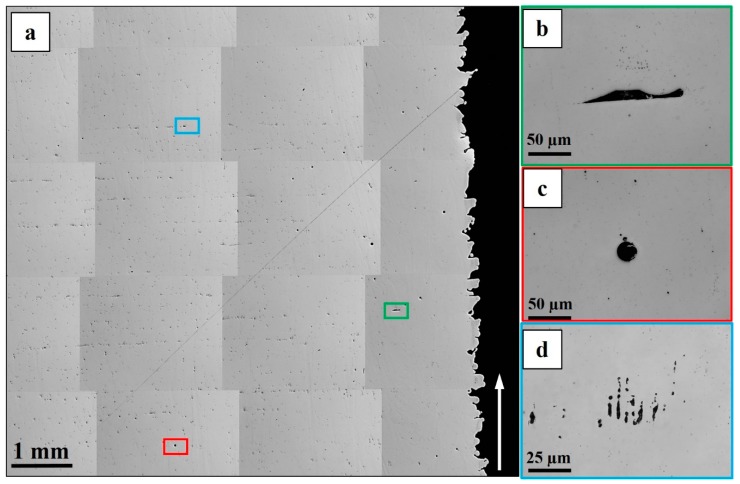
(**a**) Microstructure of the as-built material showing the different defects present. (**b**) Lack of fusion; (**c**) gas porosity; (**d**) shrinkage porosity. The arrow indicates the building direction.

**Figure 6:**
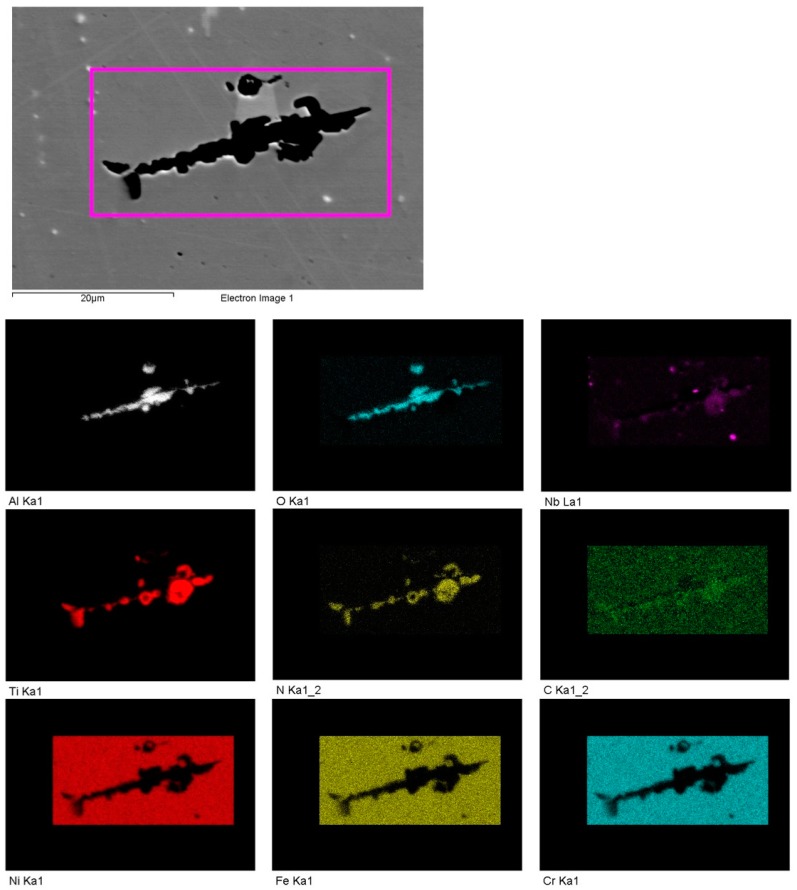
An energy dispersive x-ray spectroscopy (EDS) map around a LoF defect showing the presence of aluminium oxide and titanium nitride. Note: ‘Electron image 1′ is a backscatter electron image and the oxide and nitride appear black due to the atomic number contrast.

Three types of defects, including lack of fusion (LoF), gas porosity and shrinkage porosity, were present in the as-built condition as shown in Figure 5. These defects were distributed in a different manner within the contour, the hatch and the contour-hatch interface regions. The LoF defects were concentrated in the contour region and the contour-hatch interface region but were also present randomly in the hatch region. The shrinkage porosities, however, were concentrated in the hatch region and the contour-hatch interface region (appearing as strings aligned horizontally); very few shrinkage porosities were found in the contour region. The gas porosities were randomly distributed in all three regions. Some of these defects contained oxides of aluminium with some titanium nitride particles precipitated on the oxides. An EDS map around a LoF defect is shown in Figure 6.

**Figure 7:**
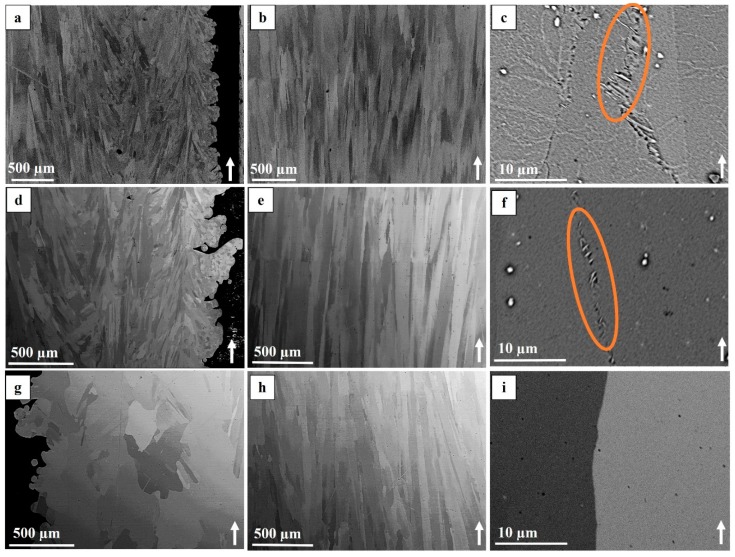
BSE images of the microstructure. (**a**–**c**) As-built condition, (**d**–**f**) STA condition, (**g**–**i**) HIP+STA condition. Images (**a**,**d**,**g**) show the contour region. All other images are from the hatch region. Images (**c**,**f**) are in etched condition; all other images are in as-polished condition. δ phase particles are indicated by the ellipses.

In the as-built condition, the microstructure in the contour was different from that of the hatch region. The contour region was composed of a combination of fine curved columnar grains elongated towards the building direction, some fine equiaxed grains and some long/large grains. The majority of the equiaxed grains were present at the periphery of the specimen, which was in contact with the powder bed. The contour-hatch interface region had columnar grains that curved towards the hatch region along the build direction. The hatch region consisted of long columnar grains typical for EBM manufactured material. Representative images showing these features are presented in Figure 7a,b. In the hatch region, some of the grain boundaries contained needle-like δ phase as shown in Figure 7c. These columnar grains also contain vertically aligned strings of carbides (NbC). The microstructure at different locations (top, middle and bottom of the specimens) was found to be identical in terms of the defects, grain morphology, NbC and δ phase distribution.

#### 3.2.2. STA Condition

**Figure 8:**
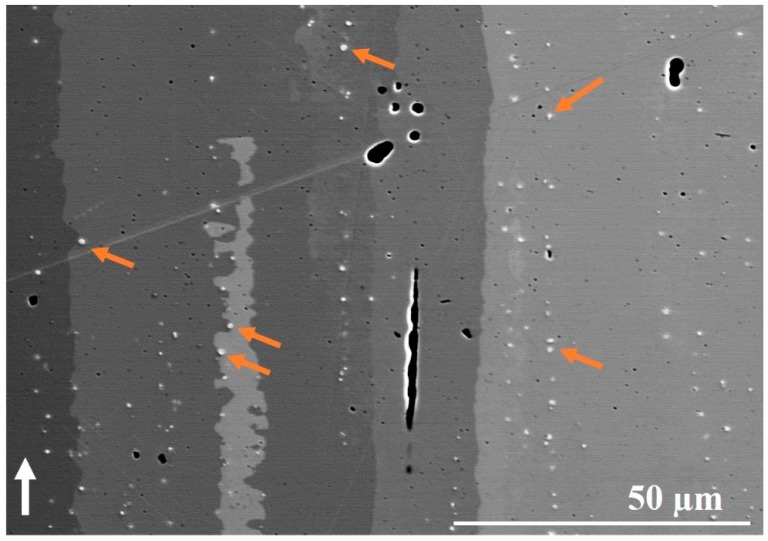
High magnification the BSE image showing strings of vertically aligned carbides. The white arrow indicates the building direction. Some of the carbide particles are indicated by arrows. Note: For the sake of brevity, the image showing carbide distribution is presented only for the STA condition.

In the STA condition, as seen in Figure 7d,e, the contour region showed the presence of fine curved columnar grains, fine equiaxed grains and some long/large grains as observed in the as-built condition; in addition, columnar grains in the hatch region and columnar grains curved towards the hatch region were also observed. The vertically aligned strings of carbides in the grains of the hatch region were also present (see Figure 8). In addition, the needle-like δ phase was found in some of the grain boundaries; however, the size of the δ phase precipitates appears to be smaller than those observed in the as-built condition (compare Figure 7c,f). Furthermore, all the defects, such as gas porosity, shrinkage porosity and LoF were also present in a distribution similar to the as-built condition.

#### 3.2.3. HIP+STA Condition

In the HIP+STA treated specimens, large equiaxed grains were present in the contour region at the periphery as shown in Figure 7g. The grain size seems to have increased, as observed in the plane parallel to the building direction, indicating that exposure to 1200 °C for 4 h in the HIP treatment results in grain growth; compare Figure 7a,d,g. In the hatch region, the columnar microstructure which has columnar grains oriented along the build direction was observed to have the same size as the other two conditions (see Figure 7b,e,h). Additionally, all of the shrinkage porosities appear to have healed. Some of the porosities and LoF defects were not fully healed, indicating that the HIP+STA post-treatment performed in this work could not eliminate the defects completely. Furthermore, in this condition, the δ phase that was observed in the as-built condition and the STA condition was not found; the carbide precipitates, on the other hand, were found in a distribution similar to the other two conditions.

#### 3.2.4. Texture

**Figure 9:**
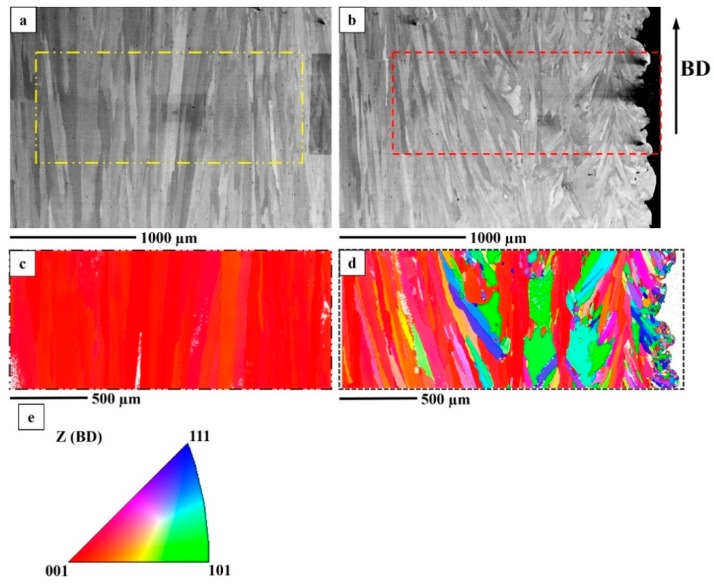
An STA specimen. (**a**) BSE image of hatch region; (**b**) BSE image of contour region; (**c**) EBSD IPF map of hatch region indicated in (**a**); (**d**) EBSD IPF map of contour region indicated in (**b**). (**e**) IPF map legend to interpret (**c**,**d**).

**Figure 10:**
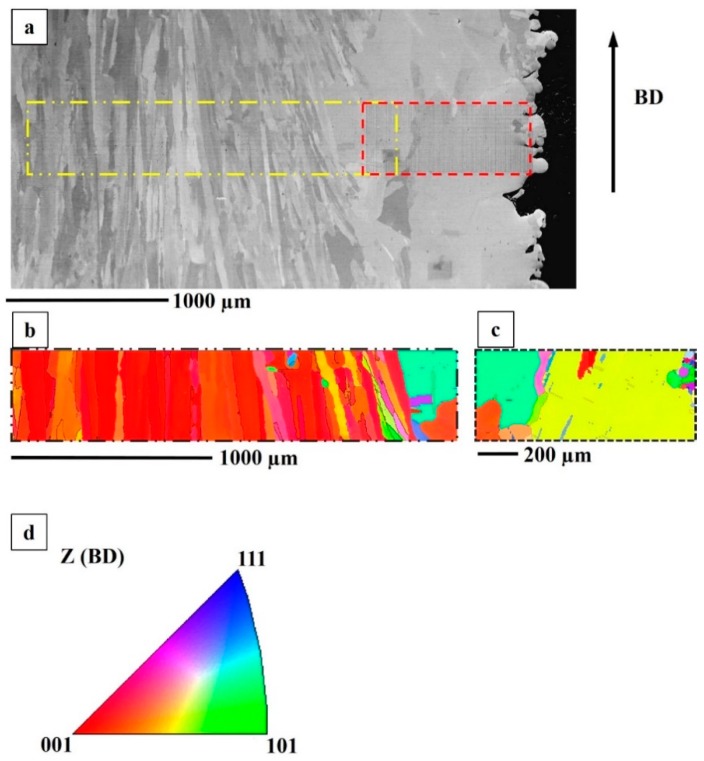
An HIP+STA specimen. (**a**) BSE image including both contour and hatch region; (**b**) EBSD IPF map of hatch region indicated in (**a**). (**c**) EBSD IPF map of contour region indicated in (**a**). (**d**) IPF map legend to interpret (**b**,**c**).

The columnar grains in the hatch region had a strong <100> texture along the building direction in the as-built condition. The contour region, which had three distinct grain morphologies, was not as textured as the hatch region. In the STA condition, a similar texture was observed pertaining to both the hatch and the contour regions, as seen in Figure 9. In the HIP+STA condition, <100> texture along the building direction was retained. The grains in the contour region were observed to have grown due to HIP treatment; however, the randomness in the orientation of grains was still present, as seen in Figure 10.

### 3.3. Hardness

**Figure 11:**
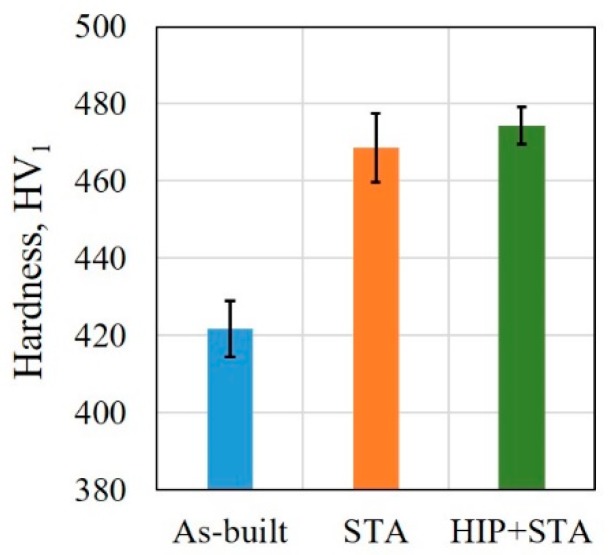
Vickers hardness (HV_1_) in the as-built and post-treated conditions.

The hardness measurements acquired from different locations of the as-built (bottom, middle and top region) and the two post-treated specimens (end 1, end 2 and middle) did not show a significant variation along the build direction. Therefore, the hardness measurements obtained from different locations of these specimens are grouped together and reported (see Figure 11). The HIP+STA and the STA specimens had higher hardness compared to the as-built specimens. The HIP+STA specimens, on average, had a slightly higher hardness compared to the STA specimens.

### 3.4. Tensile Properties

The tensile test results of the as-built, STA and HIP+STA material conditions are presented in Table 4. The yield strength (YS) and the ultimate tensile strength (UTS) in the as-built condition were inferior to both the STA and the HIP+STA condition. Specimens in both the thermal post-treated conditions had comparable mean values of YS and UTS. The highest elongation to fracture was observed for specimens in the HIP+STA condition, whereas the elongation was the lowest in the STA condition; the as-built condition showed moderate elongation.

**Figure 12:**
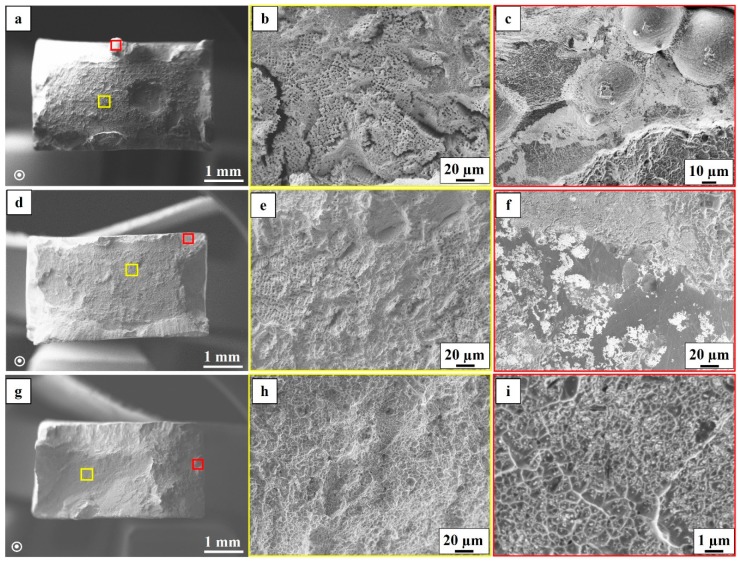
SE images of tensile fracture surfaces of (**a**–**c**) the as-built condition; (**d**–**f**) the STA condition and (**g**–**i**) the HIP+STA condition. (**b**,**e**) Shrinkage porosities; (**e**,**f**) LoF defect area. (**i**) The partially healed LoF area.

Fracture surface analysis of the ruptured specimens reveals more information on the differences in tensile properties. The fracture surfaces of specimens after tensile testing showed a typical dimpled, ductile fracture appearance, in all three material conditions (as-built, STA, HIP+STA). In the as-built and the STA conditions, shrinkage porosity and LoF defects were found, as seen in Figure 12a–f. The LoF defects often contained partially melted powder particles and an oxide film, as seen in Figure 12c,e. These defects had a detrimental effect on the tensile ductility. In the HIP+STA condition, no shrinkage porosity was observed on the fracture surface, which is in agreement with the microstructural analysis discussed earlier. The LoF defects were, in this case, found to be partially healed, that is, the LoF sites showed a fibrous appearance resembling that of dimples, with finely distributed oxide particles in between the fibrous regions (see Figure 12g–i).

### 3.5. Fatigue Properties

**Figure 13:**
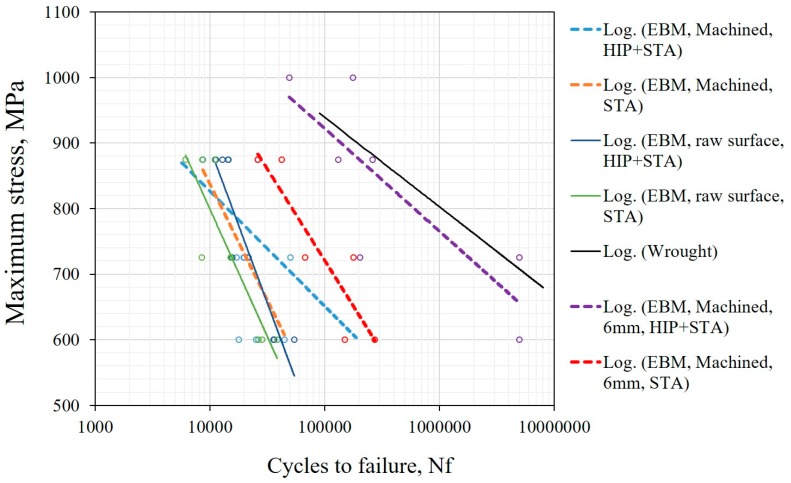
SN curves of EBM manufactured Alloy 718 in different post-treated conditions.

**Figure 14:**
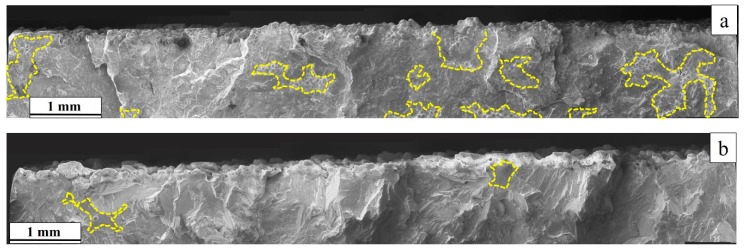
SE images of fatigue fracture surfaces showing the crack initiation area in the ‘raw’ as-built surface condition of (**a**) an STA specimen; (**b**) an HIP+STA specimen. The yellow dotted lines indicate the LoF area.

The fatigue life of EBM manufactured Alloy 718 after different thermal post-treatments, such as STA and HIP+STA in combination with different surface post-treatments such as the ‘raw’ as-built surface, the machined surface with the contour region retained (10 × 10 cross-section) and the machined surface without the contour (6 × 6 cross section) are presented in Figure 13. In all specimens with the ‘raw’ as-built surface, the fatigue life was affected by both the valley-like features in the surface and LoF defects in the contour region, as seen in Figure 14. In the specimens with machined surfaces that have 10 × 10 cross sections (in which the contour region was still present), the fatigue life was adversely affected by the presence of LoF defects. Post-treatments such as machining and HIP only marginally improve the fatigue performance and often a large scatter in fatigue life exists. The characterization of surface roughness and its effect on crack initiation, the effect of distribution and the nature of LoF defects on fatigue life have been formerly published [19].

**Figure 15:**
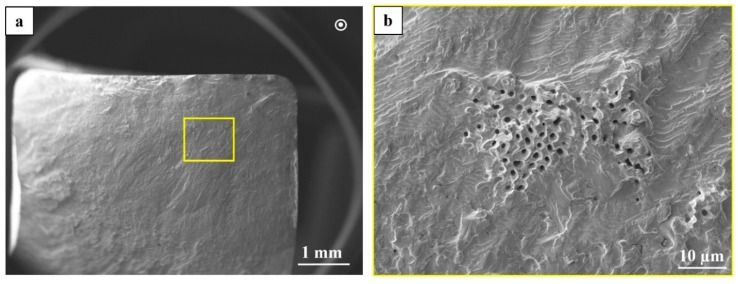
SEM images of the fatigue fracture surface of a 6 × 6 specimen in the STA condition; (**a**) overview, (**b**) shrinkage porosities in the crack propagation area.

The complete removal of the contour region by machining (for specimens with 6 × 6 cross section) improved fatigue life significantly. In the STA condition, removing the contour region improved fatigue life by approximately an order of magnitude compared to the specimens with contour, whereas in the HIP+STA condition the improvement in fatigue life is roughly two orders of magnitude. In the STA condition, shrinkage porosities are present in the crack propagation region, as seen in Figure 15. These defects were not present in the fracture surfaces of HIP+STA treated specimens. Two specimens in the HIP+STA condition, one each tested at 600 MPa and 725 MPa, sustained 5 million cycles and did not fracture. One of the specimens tested at 725 MPa failed at a relatively lower life and had fatigue crack initiation from a LoF defect on the side surface of the specimen (adjacent side to the loading side). This LoF defect had not healed because of the presence of oxides and it was not removed by machining.

**Figure 16:**
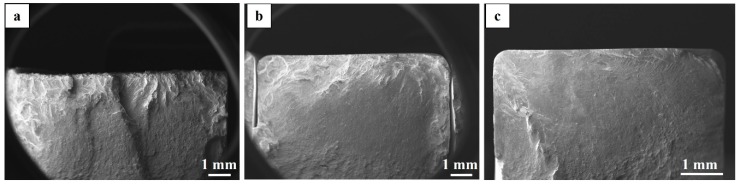
SEM images of the fracture surfaces of HIP+STA treated specimens in different surface conditions: (**a**) the ‘raw’ as-built surface, (**b**) the machined surface with the contour region and (**c**) the machined surface without contour region.

All specimens in the HIP+STA condition with the contour region (the ‘raw’ as-built specimens and machined specimens with 10 × 10 cross-section) had a faceted appearance on the fracture surface, whereas the specimens without the contour region (machined specimens with 6 × 6 cross-section) did not show a faceted appearance, as seen in Figure 16a–c. The STA specimens did not show any such faceting behaviour (see Figure 14).

## 4. Discussion

### 4.1. Powder Characteristics

The spherical particle morphology and minimal satellite particles, features of the powder used in the present study, are essential characteristics for good powder packing density and flowability. The presence of excess amounts of satellite-like features and the irregular shape of the powder particles leads to poor packing density and flowability [28]. The flowability of the powder influences how the powder is dispensed on to the powder bed, which along with the packing density could affect the build characteristics [29]. Formation of particulate oxides on the powder surface, deformation of the powder surfaces and changes in size distribution have been observed after the recycling of powders used in powder bed additive manufacturing [30,31,32]. These changes have been observed to affect the flowability of the powder. Several studies on the effects of recycling of powders and its influence on mechanical properties in powder bed additive manufacturing have concluded that the limiting factor for recycling could be oxygen pickup during the process and that not all mechanical properties are affected by the recycling [32,33,34].

### 4.2. Microstructure

The differences in the microstructure between the contour and the hatch are related to the different melting strategies. The ‘multi-spot’ strategy of the contours leads to multiple small melt pools. The steepest thermal gradient, which is locally normal to the melt pool boundary, is towards the centre of these individual spots (or short line segments). In addition, the presence of powder particles provides numerous heterogeneous nucleation sites. These new grains grow along the steepest thermal gradient and hence are curved and elongated. The long <100> textured grains are possibly at the centre of individual melt pools which form due to epitaxial grain growth from the bottom. Some of the powder particles at the periphery are only partially melted. This leads to the presence of numerous fine equiaxed grains at the periphery. The presence of other equiaxed grains could be due to random stray grain formation caused by local melt pool conditions. The re-melting of the solidified contour, due to overlap between the adjacent contour passes, leads to the formation of large grains as a result of epitaxial grain growth from the sides. The ‘snaking’ strategy in the hatch leads to the formation of a single large melt pool [35]. The steepest thermal gradient is still locally normal to the melt pool surface, which leads to the curvature of grains in the contour-hatch interface region. However, due to the rotation of the hatching direction for each layer, the highest average global thermal gradient of this large melt pool (away from the contour-hatch interface region) is along the building direction. Due to the epitaxial growth of FCC γ grains (which have the fastest crystal growth along <100> direction), the grains that have <100> orientation along the building direction are favoured. This leads to the formation of long columnar grains with strong <100> texture. Furthermore, Strondl et al. [17] reported that the texture is unaffected by thermal post-treatments, which was also observed in the present work. The grains in the contour region were found to have grown only in the HIP+STA specimens and not the STA specimens. The higher temperature of the HIP treatment (compared to the STA treatment) results in a higher diffusion rate, leading to grain growth. The reason why only the grains in the contour region have grown is unclear. The driving force for grain growth in the contour region could be to minimize the grain boundary area of the smaller grains in the region. Similar to these findings, Kirka et al. have reported that the columnar grains in the hatch region do not grow when subjected to a HIP treatment [13].

In the present study, in the as-built condition, the δ phase was found only in a few grain boundaries. Deng et al. have reported preferential precipitation of the δ phase at high angle grain boundaries in EBM manufactured Alloy 718 [9]. However, Kirka et al. have reported spurious precipitation of the δ phase in the columnar grains [10] and Unonic et al. have observed different size fractions of the δ phase at the top and bottom of the investigated specimens [36]. Such differences in the δ phase precipitation are related to thermal conditions prevailing in the specific build designs, that is, the size of the specimens, other components built together in the same build [36], build pre-heat temperature [15], spacing between the specimens, the kind of support structure used, chemical composition of the powder and cooling down after the build sequence [16]. At temperatures of ~900 °C, direct δ phase precipitation from the FCC γ matrix is also possible [37], which would hinder the precipitation of the primary strengthening phase (γ”). Since the EBM manufacturing is conducted at a higher powder bed temperature, this direct precipitation of the δ phase is also possible. It was found that the δ phase remained in certain grain boundaries even after the STA treatment in which the solution treatment temperature (1066 °C) was above δ solvus. In HIP+STA treated specimens (the material was exposed to 1200 °C) the δ phase was completely dissolved. The higher diffusion rate at higher temperatures (compared to the STA treatment) leads to complete dissolution of the δ phase in HIP+STA treated specimens. Deng et al. have reported complete dissolution of the δ phase after treatment at 1080 °C [9]. The small-sized δ phase observed in the present work found in the STA treated condition could be a result of the incomplete dissolution of the precipitates. This effect could be attributed to the local chemical variations of Nb distribution; however, further investigation is required to fully understand this.

The presence of vertically aligned strings of carbides in the interdendritic region in EBM manufactured Alloy 718 were also observed by Strondl et al. [8]. This is a result of columnar grain growth, which results in the interdendritic regions being vertical and aligned along the build direction. Since the carbides precipitate in the interdendritic regions, these manifest as strings aligned along the build direction [14]. The temperature for HIP and STA treatments used in this study are lower than the solvus temperature of NbC [38]. Therefore, the strings of carbides remain as strings and are not dissolved by any of the applied post-treatments. Kirka et al. have reported that the size of the size of the primary strengthening precipitates, γ”, can be different at the top and the bottom of the built parts in the as-built condition depending on build conditions. Furthermore, they also reported uniform size distribution of γ” after post-treatments. Though not investigated in this study, the uniformity in hardness between the different locations in the specimens in the post-treated conditions is an indication of a similar size distribution of γ”

The formation of defects and their preferential distribution stems from improper melting during manufacturing, which is related to the different melting strategies [19,39]. The shrinkage porosities in the hatch and the contour-hatch interface formed because of scan lines that were longer than optimal, which led to lack of sufficient energy for melting. In the standard theme, the process variables, such as beam current and velocity, are automatically controlled by the heat model algorithm together with several compensation functions [29]. The speed function in the theme works in a way that it adjusts the beam current and velocity proportional to the length of scan lines to keep the time for melting individual lines as constant, that is, as scan lines increase in length, both beam current and velocity are increased. However, the beam current has a fixed maximum limit and if the scan length is further increased, only the velocity of the beam increases. This results in lower energy for melting as the optimal scan length is exceeded, leading to the formation of shrinkage porosities. The LoF defects in the contour and contour-hatch interface are related to non-optimal ‘multi-spot’ melting parameters [29]. It is possible to optimize the parameters related to the contour regions to build parts with minimal LoF defects [40]. Gas porosity is randomly distributed and originates from the powder [31]. All these defects were least affected by the STA treatments, which was as expected. The HIP+STA treatment resulted in the closure of most of the defects; some LoF defects were only partially healed, which was confirmed to be related with the presence of oxides. The formation of oxides in EBM manufactured Alloy 718 has been attributed to the powder [41]. The powder used to manufacture the specimens did not have particulate oxides on the surface. Therefore, it is possible that the oxides observed on the fracture surfaces were formed during the EBM process by transformation of the passive oxide layer in the powder and/or by oxidation in the chamber [41].

### 4.3. Hardness

The post-treated specimens showed higher hardness than the as-built specimens, as expected due to the precipitation hardening in Alloy 718 resulting from the applied ageing treatment. Comparing the HIP+STA and STA specimens, the HIP+STA specimens showed slightly higher hardness which is attributed to the closure of defects such as shrinkage porosity and LoF by the applied HIP treatment [42]. The difference, however, is not high enough to suggest that the HIP+STA treatment will lead to a higher hardness than the STA treatment alone. Strondl et al. [17] reported that there is no difference in hardness between the top and bottom of the builds, which was also observed in the present study. Deng et al. [9] reported similar hardness values to that observed in the current work for the as-built and post-treated EBM manufactured Alloy 718.

### 4.4. Tensile Properties

The YS and UTS of the specimens in both post-treated conditions were higher than in the as-built specimens. This increase in YS and UTS is expected after an ageing treatment, after which the precipitation of the γ” phase occurs. The elongation to fracture, however, was higher in the HIP+STA treated specimens than in the as-built specimens. Since defects were healed (or partially healed in case of LoFs, see Figure 12i) after HIP+STA treatment, these specimens showed higher elongation to failure. This was also evident from the fractographic analysis. The STA treated specimens, on the other hand, showed lesser elongation to failure than the as-built specimens, even though defects were present in both the cases. The STA treatment does not affect the defects but leads to an increase in strength; this increase in strength makes the material more notch sensitive, which manifests in a drop of ductility. Strondl et al. reported that shrinkage porosities are responsible for the lesser elongation to fracture in EBM manufactured Alloy 718 [17]. The Young’s modulus values measured parallel to the build direction were comparable for the as-built and post-treated conditions. Additionally, the modulus values obtained in this work are comparable to those reported by Körner et al., Deng et al. and Kumara et al. using experimental and modelling studies for EBM manufactured Alloy 718 [9,43,44]. The low Young’s modulus of ~140 GPa measured along the build direction are attributed to the fact that the <100> direction is the softest direction in FCC crystals. A similar effect of the lower Young’s modulus of ~160 GPa is also reported for SLM manufactured and post-treated Alloy 718 specimens due to the <100> texture [24].

The tensile properties measured in this work for both post-treated conditions (STA and HIP+STA) are higher than typical properties of cast Alloy 718; however, the wrought form of Alloy 718 has still higher UTS. The differences in the mechanical properties between these different material forms is attributed to differences in grain size, texture, defects, the condition of precipitate phases and so forth [45,46]. Furthermore, for the HIP+STA condition, the YS achieved in this work is slightly higher than that reported by Kirka et al. for EBM manufactured and HIP+STA post-treated Inconel 718 [25]. However, the UTS and elongation values achieved in this work were slightly lower compared to those reported by Kirka et al. [25]. SLM manufactured and post-treated Alloy 718 material is often reported to have higher YS and UTS values than the EBM manufactured material with similar or higher ductility [24,25,47]. In addition, anisotropy in total plastic strain accumulation during LCF testing of EBM manufactured Alloy 718 has also been reported [48]. Therefore, a detailed investigation of the deformation behaviour of EBM manufactured Alloy 718 is necessary to fully understand and explain the mechanical behaviour.

### 4.5. Fatigue Properties

The stress concentrations in the vicinity of both the *valley like* features in the ‘raw’ as-built surface and the LoF defects promote crack initiation and thus result in poor fatigue performance of the 10 × 10 specimens. A detailed analysis of the effect of as-built surface and LoF defects on fatigue life has been presented elsewhere [19]. These features, which were in the contour and contour-hatch interface region, were present at/near the maximum stressed region under the bending load conditions. Therefore, these defects have an even larger influence on the fatigue properties than the hatch region.

The complete removal of the contour region ensured removal of the LoF defects, which thereby improved the fatigue strength significantly. In this case, only the hatch region influenced the fatigue properties. In these small 6 × 6 specimens that were STA treated, shrinkage porosities affect crack propagation, possibly by the local acceleration of crack growth rate when the crack front reaches these defects. This was evident from the fracture surfaces that showed the presence of shrinkage porosities in the crack propagation region, as shown in Figure 15. The closure of shrinkage porosities due to HIP+STA treatment had a positive effect on crack propagation behaviour by eliminating the local crack growth rate acceleration associated with these defects. Due to the compounding effect of mitigating LoF defects and shrinkage porosities, that is, improved crack initiation and propagation resistance, the HIP+STA treated 6 × 6 specimens showed fatigue life that was almost two orders of magnitude higher.

In coarse-grained material, fatigue crack growth can happen via cyclic cleavage mode due to the single shear mechanism [49]. Furthermore, the cyclic hardening behaviour of textured material is different from that of non-textured polycrystalline material [49]. As described earlier, the grains in the contour region of the HIP+STA specimens were coarsened and were not textured along the build direction. In addition, the grains in the 6 × 6 specimens were all <100> textured and were not coarsened. Therefore, differences in deformation behaviour and crack propagation behaviour due to the difference in both the size and the texture of grains at/near the maximum stressed region of the specimens could explain the faceting behaviour observed in the fracture surfaces of the HIP+STA specimens. Similarly, the absence of faceting in STA specimens can be related to the randomly oriented finer grain structure. Hence, it is possible that the <100> texture of the EBM manufactured material influences both the crack initiation and crack propagation behaviour. As noted earlier, anisotropic total plastic strain accumulation in LCF testing has been reported for EBM manufactured and HIP+STA treated Alloy 718 [48]. Thorough investigations on the deformation behaviour of EBM manufactured Alloy 718 are necessary to explain the influences of texture and grain size on the mechanical properties.

## 5. Conclusions

In this work, EBM manufactured Alloy 718 was investigated in the as-built condition and in two thermal post-treated conditions, STA and HIP+STA. Mechanical properties such as hardness, tensile and fatigue properties were evaluated. From this study, it can be concluded that:

- In the as-built condition, the needle-like δ phase was present only at certain grain boundaries. In the STA condition, the δ phase precipitates were smaller in size than those observed in the as-built condition. In the HIP+STA condition, the δ phase was not observed at all due to complete dissolution.
- The HIP+STA treatment resulted in grain coarsening in the contour region, while no change in grain size was found after the STA treatment when compared to that of the as-built material.
- The hardness of the as-built material was lower than that of the STA and HIP+STA treated materials. The material in the HIP+STA condition was marginally higher than in the STA condition.
- The post-treatments led to an increase in YS and UTS. HIP+STA led to improved elongation compared to STA due to the closure of defects but YS and UTS were comparable in both the conditions. Fracture analysis of HIP+STA specimens showed partial healing of LoF defects.
- Fatigue strength improved with the HIP+STA treatment. The highest fatigue strength was achieved when the contours were completely removed prior to testing.
- The faceted appearance of fatigue fracture surfaces was found to be affected by both the grain size and the texture.

## Figures and Tables

**Table 1:** The chemical composition of Alloy 718 powder used to manufacture the specimens investigated in this study.

**Element**	**Ni**	**Cr**	**Nb+Ta**	**Mo**	**Co**	**Ti**	**Al**	**Mn**
wt %	52.54	18.9	4.9	2.97	<0.01	0.98	0.47	0.18
**Element**	**Si**	**Cu**	**C**	**P**	**S**	**B**	**Ta**	**Fe**
wt %	0.04	0.0	0.03	<0.001	0.001	<0.001	<0.01	bal.

**Table 2:** Process parameters in the Inco 4.2.76 theme for electron beam melting (EBM) manufacturing of Alloy 718.

Parameter	Outer Contour	Inner Contours	Hatch
Max. melt current (mA)	8	8	18
Speed function	6	30	63
Focus offset (mA)	3	3	15
No. of spots [multi-spot]	40	40	N/A
Spot on time (ms) [multi-spot]	0.6	1.1	N/A
Spot overlap (mm) [multi-spot]	0.3	0.2	N/A
Layer thickness (µm)	75		
Hatch rotation (degree)	72		
Pre-heat temperature (°C)	1025		
Line off-set in hatch (mm)	0.125		
Off-set between hatch and contour (mm)	0.2		
Off-set between contours (mm)	0.3		

**Table 3:** Specimen types for fatigue testing.

Thermal Post-Treatment	Surface Post-Treatment	Cross-Section (mm^2^)	No. of Specimens
HIP+STA	As-built	10 × 10	9
HIP+STA	Machined	10 × 10	9
HIP+STA	Machined	6 × 6	6
STA	As-built	10 × 10	9
STA	Machined	10 × 10	9
STA	Machined	6 × 6	6

**Table 4:** Tensile properties of EBM manufactured Alloy 718.

Material Condition	Yield Strength (MPa)	Ultimate Tensile Strength (MPa)	Elongation (%)	Young’s Modulus (GPa)
As-built	920 ± 16	1075 ± 46	10 ± 3	138 ± 5
STA	1096 ± 6	1172 ± 30	6 ± 1	137 ± 7
HIP+STA	1100 ± 13	1190 ± 33	14 ± 1	142 ± 4
Cast (AMS 5383) [26]	≥ 760	≥ 860	≥ 5	-
Wrought (AMS 5662) [27]	≥ 1034	≥ 1275	≥ 12	-

## Data Availability

The raw/processed data required to reproduce these findings cannot be shared at this time as the data also forms part of an ongoing study.
